# Building a case for ‘functional identity fraud’ in eRpL22 paralogue-specific ribosomes in *Drosophila* germline development

**DOI:** 10.1098/rstb.2023.0391

**Published:** 2025-03-06

**Authors:** Vassie C. Ware

**Affiliations:** ^1^ Department of Biological Sciences, Lehigh University, Bethlehem, PA 18015, USA

**Keywords:** ribosomal protein paralogues, *Drosophila* spermatogenesis, eRpL22 and eRpL22-like

## Abstract

Investigations of expression and function of eukaryotic-specific ribosomal protein paralogues, eRpL22 and eRpL22-like, within the *Drosophila melanogaster* male germline offer valuable insights supporting an emerging paradigm shift that ribosomes are now exempt from the traditional view of being homogeneous protein synthesis machines. Co-expression of these paralogues within the same cell contributes to structural and functional complexity—the latter demonstrated by differential translation specificities based on paralogue content. This commentary highlights some of the key findings related to the biology of specialized ribosomes containing paralogue eRpL22 or eRpL22-like in *Drosophila* spermatogenesis and raises several unresolved questions about eRpL22 family paralogue function and ribosome-mediated translation regulation within spermatogenesis. Our understanding of principles that govern specialized ribosome function is in nascent stages, and considerably more research is warranted to address the myriad of unresolved questions about specialized ribosomes and the impact on reproductive physiology.

This article is part of the discussion meeting issue ‘Ribosome diversity and its impact on protein synthesis, development and disease’.

## 1. Introductory perspective

A rationale for exploring the functional significance of ribosomes differing in molecular composition or biochemical character has gained considerable traction, particularly as evidence mounts that ‘specialized ribosomes’ display different translational specificities in development or cellular differentiation (e.g. reviewed in [1–6]). Studies of co-expression of ribosomal protein (RP) paralogues eRpL22 and eRpL22-like in the *Drosophila melanogaster* male germline are an ideal model exploring mechanisms whereby ribosome heterogeneity at the RP level could dictate differences in ribosome function within the same cell at different stages of sperm cell differentiation.

Our studies and those of others have contributed important findings on the role of eRpL22 family paralogues in spermatogenesis. A strong argument for functional diversification between *D. melanogaster* eRpL22 paralogues and orthologues from other eukaryotes could be offered simply based on significant protein structural differences, notably within the N-terminal extension that has homology to the histone H1 C terminus [7,8]. Further, the N-terminal extension between fly eRpL22 and eRpL22-like reveals only 36% amino acid (aa) sequence identity and 47% aa sequence similarity [8]. While both paralogues are essential [9–11], differences in the severity of developmental phenotypes resulting from paralogue depletion provide additional hints of divergent functional roles for eRpL22 and eRpL22-like in development. Depletion of either paralogue has lethal ramifications at different stages of development. Disruption of eRpL222 results in embryonic lethality but variation in timing and degree of eRpL22-like depletion results in phenotypes affecting several organ systems, including testis development and subsequent production of mature sperm [11].

More definitive support for the functional diversification hypothesis for fly eRpL22 family paralogues has come from more recent molecular data. Analyses of testis RNA sequencing data revealed the first *in vivo* evidence that testis ribosome heterogeneity impacts translation regulation, showing enrichment of different mRNA populations on eRpL22 or eRpL22-like polysomes in spermatogenesis [12]. The physiological impact of differential association of specific mRNAs with paralogue-specific ribosomes during sperm maturation is unknown. However, changes in sperm maturation and fertility have been documented after constitutive RNAi-mediated depletion of eRpL22 and overexpression of eRpL22-like (collectively , which restores embryonic viability and developmental progression). Under these circumstances, late stages of sperm maturation and fertility in adult flies are compromised [11]. Whether or not deficiencies in the testis proteome (resulting from perturbations in specific mRNA translation owing to an imbalance in RpL22 and eRpL22-like ribosome levels under these circumstances) account for observed fertility defects remains to be determined.

**Figure 1.**
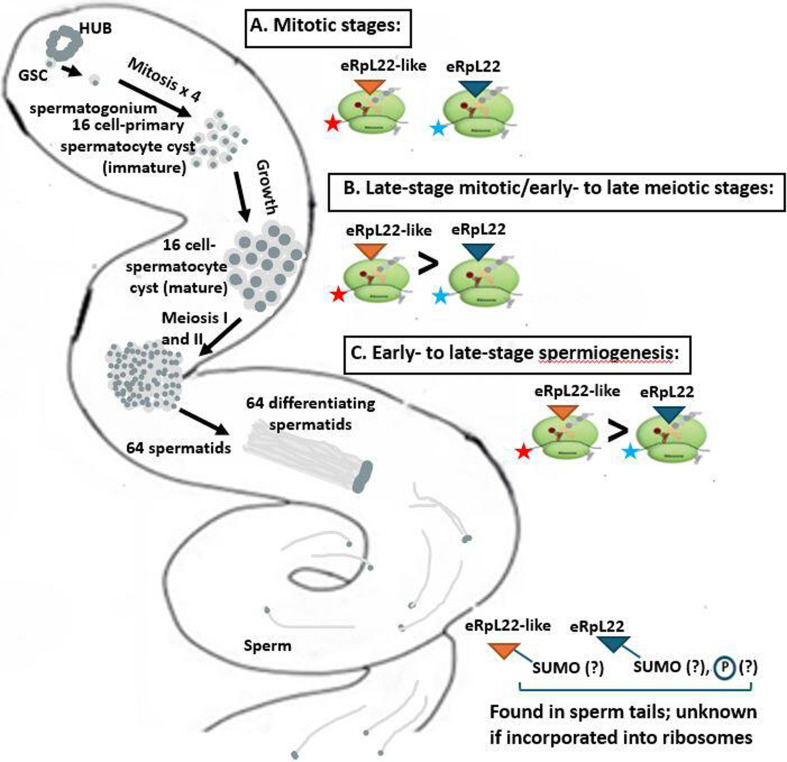
A working model for eRpL22 and eRpL22-like paralogue functional divergence and ribosome heterogeneity in *Drosophila* spermatogenesis. (A) Mitotic stages. Both paralogues are ribosomal components. eRpL22 is expressed in both somatic cyst cell lineages and germ cells [8], but eRpL22-like expression is confined to the germline, yielding eRpL22 family heterogeneity within the germline ribosomal pool. eRpL22 is a substrate for post-translation modifications (PTMs), including SUMOylation (SUMO) and a pattern of testis-specific phosphorylation [13]. SUMOylated eRpL22 is excluded from the translation machinery and may act extraribosomally [13]. Some mRNAs (depicted with red and blue stars) are enriched and translated on eRpL22- or eRpL22-like-specific ribosomes ([12]; see §§2a-c for further discussion of translation specificities of eRpL22- and eRpL22-like-ribosomes throughout spermatogenesis stages, A–C). (B) Late-stage mitotic/early to late meiotic stages. Demonstrated through quantitative tandem mass spectrometry, the proportion of eRpL22-like ribosomes exceeds that of eRpL22 ribosomes through paralogue switching [14]. The distribution of eRpL22 changes from a cytoplasmic pattern (where ribosome function occurs) to a primarily nucleoplasmic, punctate pattern in primary spermatocytes [13,15]. Germ cell-specific PTMs occur after G2/M of meiosis I ([13]; MG Kearse and VC Ware, unpublished data, 2013). This predominant eRpL22 subcellular re-distribution correlates with PTM changes [13] and measurements of eRpL22 and eRpL22-like in ribosomes by Hopes *et al*. [14]. We do not yet have a comprehensive view of possible nuclear extraribosomal roles for SUMOylated eRpL22, but this includes inter-regulation of eRpL22/eRpL22-like paralogues to ensure proper RP dosage ([15]; see §2b for further discussion). (C) Early- to late-stage spermiogenesis. The presence of at least a small pool of eRpL22 ribosomes is supported by the preferential association of several known post-meiotically transcribed mRNAs (e.g. *cup* and *comet* mRNAs) on eRpL22 polysomes [12]. Both paralogues localize within sperm tails [8]. eRpL22-like is likely SUMOylated in mature sperm (CM Mageeney and VC Ware, unpublished, 2018). Preservation of specific eRpL22 PTMs and the ribosomal status of both paralogues are unknown within mature sperm.

A working model for paralogue functional divergence and ribosome heterogeneity within the testis is shown in figure 1. Briefly, in *D. melanogaster* spermatogenesis (reviewed by Fuller [16]) from the tip of the testis at the hub, a spermatogonium emerges from the germline stem cell niche to undergo four mitotic divisions, with incomplete cytokinesis. Two terminally differentiated somatic cyst cells surround the dividing spermatogonium during mitosis to the 16-cell stage and throughout meiosis to eventually yield 64 spermatids. Dramatic morphological remodelling transforms round spermatids into elongated, mature sperm reminiscent of *ca* 2 mm-long ‘javelins’. An intricate program of gene expression unfolds during this process, during which eRpL22 and eRpL22-like have vital and partially inimitable roles, based on their inability to completely substitute for functions provided by the opposing paralogue [11]. At key developmental stages of spermatogenesis, critical insights from studies of eRpL22 paralogues are highlighted. Further, several unresolved questions related to possible differential roles of eRpL22 family paralogues in stages of spermatogenesis are proposed for consideration.

## 2. Key roles for eRpL22 and eRpL22-like paralogues in spermatogenesis

### (a) Mitotic stages of spermatogenesis

Intricate patterns of regulated transcription and translation unfold during spermatogenesis to produce mature sperm. Overall, transcription is downregulated in primary spermatocytes [17], while genes encoding testis-specific products (many of unknown function) are expressed during mitotic stages (reviewed by Schäfer *et al*. [18]). Our understanding of the role of ribosome-mediated regulation in unfolding the program of gene expression in spermatogenesis remains limited.

Investigations of mRNA content of specific antibody-purified eRpL22- and eRpL22-like polysomes provide insights into proposed specialized functions for each ribosome type in establishing the testis proteome. While there is some overlap in the translatomes of paralogue-specific ribosomes, nearly 50% of mRNAs are selectively translated on eRpL22- or eRpL22-like polysomes [12]. eRpL22 ribosomes translate *early* testis-specific and ubiquitously expressed transcripts, while eRpL22-like ribosomes primarily translate testis-specific transcripts throughout all stages of spermatogenesis. Overall, translation specificities are influenced by RP paralogue composition within the ribosomal pool at specific stages of spermatogenesis. Identification of six testis-specific RP paralogues, including eRpL22-like, within testis 80S ribosomes [14] interjects additional layers of ribosome composition complexity that may specify different functional capacities for 80S ribosomes with different combinations of paralogues. Development of strategies to isolate 80S ribosomes comprised of eRpL22 or eRpL22-like and another testis-specific paralogue type, with subsequent mRNA profile analyses, will extend our understanding of the degree to which specialized ribosomes based on paralogue composition influence the testis proteome.

Several additional questions emerge after consideration of the impact of paralogue composition on ribosome function and the ribosomal pool in mitotic stages of spermatogenesis. How are specific mRNAs targeted to eRpL22- or eRpL22-like-specific ribosomes? What molecular signatures on mRNAs and ribosomes are crucial ‘guides’ for mRNA association with specific ribosome types? What is the role of SUMOylated eRpL22 in pre-meiotic spermatocytes? Is newly synthesized eRpL22 SUMOylated and then excluded from incorporation into ribosomes or is eRpL22 SUMOylated *in situ* on ribosomes and exchanged for unmodified eRpL22 or eRpL22-like? Pursuit of answers to these questions will expand our view of the impact of ribosome heterogeneity on the establishment of the proteome in spermatogenesis.

### (b) Late-stage mitotic/early- to late meiotic stages

Based on differential mRNA association with specific ribosome types, we predict that changes in the proportion of ribosome types (based on RP composition, for example) within the ribosomal pool ultimately influence the pattern of mRNA expression throughout spermatogenesis. An outcome of SUMOylation of eRpL22 is that changes arise in the makeup of the ribosomal pool in meiotic spermatocytes occur, although direct causal links have not been established [13]; the proportion of eRpL22 ribosomes relative to eRpL22-like ribosomes in the testis is reduced (as measured by Hopes *et al*. [14]). In principle, changes in eRpL22 ribosome availability would alter the translation of early testis-specific transcripts preferentially associated with eRpL22 ribosomes and be part of a complex mechanism to regulate levels of early testis-specific products at meiotic stages.

Little is known about the function(s) of SUMOylated eRpL22, but recent studies by Ng *et al*. [15] demonstrate that SUMOylated eRpL22 binds to a stable intronic sequence RNA called *circ*RpL22, which is derived from the eRpL22 locus. This circRNA plays a role in crosstalk between eRpL22 and eRpL22-like to regulate levels of each RP, particularly at earlier stages of spermatogenesis. Binding of SUMOylated eRpL22 to circRpL22 facilitates repression of the *eRpL22-like* gene and has a role in the autoregulation of eRpL22 expression. Overall, mechanisms that control eRpL22 localization and that regulate levels of eRpL22 and eRpL22-like into ribosomes within specific stages of spermatogenesis are largely unknown and await additional detailed investigations.

### (c) Early- to late-stage spermiogenesis

Once meiotic germ cells are on the path to differentiation from round spermatocytes to elongated, mature sperm, dramatic changes in transcription and translation occur. Post-meiotic transcription in early spermatids is significantly reduced [17]. Within mid-to-late elongating spermatids, post-meiotic transcription of *comet* and *cup* genes is reactivated with the deposition of these mRNAs at the growing ends of spermatids [17]. Translation of several translationally silenced mRNAs occurs at this stage of spermatogenesis [18]. Translation of several mRNAs and some late-stage testis-specific mRNAs occurs on eRpL22-like ribosomes [12]. Interestingly, some post-meiotically transcribed mRNAs (including several *cup* and *comet* mRNAs) are translated on eRpL22 ribosomes, demonstrating the presence of a translationally active population of eRpL22 ribosomes at this late stage of spermatogenesis. The presence of *comet* and *cup* mRNAs at the growing ends of spermatids suggests that eRpL22 ribosomes may be localized to these regions to facilitate translation. It is unknown whether *comet* and *cup* mRNAs are stored on eRpL22 ribosomes in an inert state until the release of the translation block in late-elongating spermatids. It is also unknown whether there is a population of specialized eRpL22 ribosomes that are sequestered in the early stages of meiosis that function in the later stages of spermatogenesis. Finally, within mature motile sperm, the PTM and ribosomal status of eRpL22 and eRpL22-like are unknown. With evidence that eRpL22-like is SUMOylated at this stage (CM Mageeney and VC Ware, unpublished, 2018), it may be that SUMOylated eRpL22-like is excluded from ribosomes in a similar fashion as in the case of SUMOylated eRpL22. Yet, if paralogues at this stage of spermatogenesis are ribosomal components, complete entry of the entire 1.8 mm *D. melanogaster* sperm flagellum into the egg during fertilization ([19]; reviewed by Loppin *et al*. [20]) offers an intriguing idea for how specialized ribosomes may influence embryonic development in the next fly generation. Even if paralogues are not in ribosomal complexes at this stage, entry of eRpL22 and eRpL22-like into the egg cytoplasm ‘delivers’ the prospect for functional impacts in the newly fertilized egg.

## 3. Closing arguments

It is highly likely the work of several research groups studying the role of eRpL22 and eRpL22-like in the specialized ribosome story in *Drosophila* spermatogenesis will gain further insights into the role of ribosome-mediated regulation in reproductive physiology and developmental processes in other systems, including humans. For eRpL22 or eRpL22-like, its position on the large subunit surface warrants consideration of what structural features of the paralogue itself and interactions with other ribosomal and/or ribosome-associated components are required to enable differential association of mRNAs to specific ribosome types. It is crucial to understand how heterogeneity in the ribosome pool, contributed by changes in RP paralogue content at different stages of the sperm maturation pathway, impacts the programme of gene expression for normal development and how disruption of that pathway could facilitate fertility defects. Broader impacts from studies of the eRpL22 family in *Drosophila* may include the emergence of generalized concepts for how ribosome heterogeneity and specialization contribute to the complexities of normal development and disease states.

## Data Availability

This article has no additional data.
